# Deficient Signaling via Alk2 (Acvr1) Leads to Bicuspid Aortic Valve Development

**DOI:** 10.1371/journal.pone.0035539

**Published:** 2012-04-19

**Authors:** Penny S. Thomas, Somyoth Sridurongrit, Pilar Ruiz-Lozano, Vesa Kaartinen

**Affiliations:** 1 Department of Biologic and Materials Sciences, University of Michigan, Ann Arbor, Michigan, United States of America; 2 Department of Pediatrics, Stanford University School of Medicine, Stanford, California, United States of America; Childrens Hospital Los Angeles, United States of America

## Abstract

Bicuspid aortic valve (BAV) is the most common congenital cardiac anomaly in humans. Despite recent advances, the molecular basis of BAV development is poorly understood. Previously it has been shown that mutations in the *Notch1* gene lead to BAV and valve calcification both in human and mice, and mice deficient in *Gata5* or its downstream target *Nos3* have been shown to display BAVs. Here we show that tissue-specific deletion of the gene encoding Activin Receptor Type I (*Alk2* or *Acvr1*) in the cushion mesenchyme results in formation of aortic valve defects including BAV. These defects are largely due to a failure of normal development of the embryonic aortic valve leaflet precursor cushions in the outflow tract resulting in either a fused right- and non-coronary leaflet, or the presence of only a very small, rudimentary non-coronary leaflet. The surviving adult mutant mice display aortic stenosis with high frequency and occasional aortic valve insufficiency. The thickened aortic valve leaflets in such animals do not show changes in Bmp signaling activity, while Map kinase pathways are activated. Although dysfunction correlated with some pro-osteogenic differences in gene expression, neither calcification nor inflammation were detected in aortic valves of *Alk2* mutants with stenosis. We conclude that signaling via Alk2 is required for appropriate aortic valve development in utero, and that defects in this process lead to indirect secondary complications later in life.

## Introduction

Bicuspid aortic valve (BAV) is the most common cardiac malformation affecting 1–2% people worldwide [1]–[3]. While those with BAV often remain asymptomatic for years, almost all will eventually require surgical intervention [3]. The molecular mechanisms underlying BAV formation are still poorly elucidated. In humans, mutations in the *NOTCH1* gene have been shown to lead to BAV [4]. An increased incidence of BAV has also been found in in mice deficient in the transcription factor Nkx2-5 [5], endothelium-specific nitric oxide synthase *Nos3* [6] and, most recently, transcription factor Gata5, which is proposed to positively regulate *Nos3* expression and affect the Notch signaling pathway [7].

An initial key event in cardiac valve development is formation of endocardial cushions [8], which starts with a regional increase of cardiac jelly, a hyaluronic acid-rich extracellular matrix (ECM), between the myocardial and endocardial cell layers at the sites of the future atrio-ventricular junction (AVJ) and outflow tract (OFT) [9]. This is followed by transformation of a subset of the overlying endocardial cells to mesenchymal cells (EMT) [10] which migrate into the underlying ECM to populate the cushions. In the OFT, the proximal cushion mesenchyme is so derived from the endocardium, and much of the more distal cushion mesenchyme is of cranial neural crest origin [11]. Before OFT septation, four ridge-like cushions spiral around the internal circumference of the OFT lumen; two larger (septal and parietal) and, between them, two smaller (anterior and posterior intercalated). The two larger cushions fuse across the midline of the OFT lumen, creating separate outlet lumens connecting the left and right ventricles to their respective arterial valves (aortic and pulmonary). Around each of these, further cushion growth from each of the larger (fused) cushions and the intercalated cushion forms the three arterial valve leaflet precursors for each truncal valve (aortic and pulmonary) [12]. After further development, the right and left coronary arteries connect to the aortic valve sinuses in characteristic positions immediately downstream of the right and left coronary leaflet precursors respectively [13]. The remaining leaflet, the ‘non-coronary’, is the one arising from the intercalated cushion position. These cushions undergo characteristic morphogenesis to form three flexible apposing leaflets and adjacent sinuses [14]. Normal function of these leaflets requires that they are sufficiently apart during ventricular systole, and appose each other to prevent backflow during diastole, otherwise aortic stenosis and insufficiency respectively will result. A critical distinctive extracellular matrix arrangement is required to maintain these properties throughout life [15]–[17]. With time, or if the leaflets are abnormal in shape or number, the leaflets are more likely to fail mechanically and develop abnormal structure and function [18], [19]. From examination of human examples, a reduction in effective leaflet number to form a bicuspid (two leaflet, or bifoliate) aortic valve (BAV) has been proposed to arise to from fusion of two leaflets, including before birth at the ‘cushion’ stage, rather than the presence of only two leaflet cushions per arterial valve at the outset.

In humans, anatomical classification of BAVs indicates all combinations of leaflet fusion can be found, the two most common being right- with non-coronary leaflets (R-N), and right with left-coronary leaflets (R-L) [3], [20]. A recent comparative study of the *Nos3* knockout mouse (R-L) and inbred Syrian hamster (R-L) bicuspid models demonstrates that the etiologies of these phenotypes are different [21]. Given that patients with R-N leaflet fusion develop different clinical phenotypes from those of patients with R-L leaflet fusion, mechanistic understanding of aortic valve development at both morphological and molecular levels is of critical importance [22], [23].

Bmp signaling has been shown to play a critical role during cardiac valve development. Several studies have shown that Bmp2 is able to induce EMT both in vitro and in vivo [24], [25]. Both Bmp type I receptor Alk3 (Bmpr1a) and Activin type I receptor (Alk2; Acvr1) are expressed in the endocardium, and epithelium-specific deletion of these receptors leads to EMT defects in both OFT and AV cushions [25]–[28]. The importance of Alk2-mediated signaling in human cardiac disease is highlighted by recent studies, which demonstrate that mutations in the *ALK2* gene are also responsible for cardiac defects in humans [29], [30].

While the role of *Alk2* in endothelial EMT is well-established [26], very little is known about Alk2-mediated Bmp signaling in subsequent stages of valve development. In this study we examined the role of Alk2 in cardiac valve development by deleting *Alk2* function in the cushion mesenchyme after EMT using the *Gata5-Cre* driver line. Most of the resulting *Alk2* mutant mice developed aortic valve defects characterized as ‘functional’ BAV as they consisted of only two, apposed, morphologically functional aortic leaflets (or their precursors), even if other structures were present, prior to birth. A subset of mutant mice that survived to adulthood developed aortic insufficiency and/or stenosis associated with some markers of a pro-osteogenic phenotype: attenuated *Sox9* expression, increased *osteopontin (Spp1)* expression and activated Erk1/2 but did not develop calcification. These results suggest that signaling via Alk2 is required for normal aortic valve development and that defects in this process lead an increased incidence of aortic insufficiency and/or stenosis and formation of a bicuspid aortic valve phenotype commonly found in humans.

## Results

### Gata5-Cre drives an efficient recombination in the endocardial cushion mesenchyme

**Figure 1:**
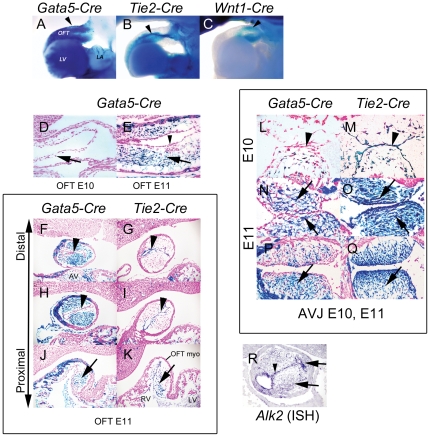
The *Gata5-Cre* transgene induces recombination in the endocardial cushion mesenchyme after EMT. Comparison of areas of *Gata5-Cre (A), Tie2-Cre* (B) and *Wnt1-Cre* (C) recombination (blue) in OFT region (arrowhead) at E11.5 (wholemount *R26R*-driven βgal stain, viewed from the left side, distal OFT to the right). βgal staining of sagittal sections at E10 (D) and at E11 (E) shows extensive *Gata5-Cre*-induced recombination in OFT cushion mesenchyme by E11 (arrow), but not in endocardium (arrowhead). Comparison of *Gata5-Cre* (F, H and J) and *Tie2-Cre* (G, I and K) recombination patterns (*R26R*-driven β-gal stain in blue) at three levels of the OFT, from proximal (J, K) to distal (F, G). *Tie2-Cre* (arrowheads, G, I) but not *Gata5-Cre* (arrowheads F, H) drives recombination (blue) in endocardium; recombined mesenchymal cells (blue) in both lines (arrows, J, K) only in proximal OFT. Comparison of *Gata5-Cre* and (L, N and P) *Tie2-Cre* (M, O and Q) recombination at AV junction: sagittal sections (L–O) at E10 (L, M) and E11 (N, O) and transverse sections at E11 (P, Q). At E10 (L, M) endocardium (arrowhead) recombined (blue) by *Tie2-Cre* but not *Gata5-Cre*. At E11 (N–P) AV mesenchymal cells (arrows) recombined (blue) by both *Tie2-Cre* and *Gata5-Cre*. R, In situ hybridization for *Alk2* RNA showing expression (blue) in OFT cushion mesenchyme (black arrows) and endocardium (arrowhead) at E11.5. LV, left ventricle; RV, right ventricle; OFT myo, OFT myocardium; AV, AV cushion; AVJ, atrioventricular junction.

The *Gata5-Cre* transgenic mouse line was originally designed as an epicardium-specific driver line [31]. When analyzing recombination patterns in these mice, we noticed that in addition to epicardial cells and occasional myocardial cells, efficient recombination could also be detected in endocardial cushion mesenchyme at both the OFT and AV junction at E11 (Figure 1). Unlike the *Wnt1-Cre* and *Tie2-Cre* drivers, which induce recombination in neural crest (NC) cells including the distal cushion mesenchyme, and in endothelial cells and endocardium-derived proximal cushion mesenchyme, respectively, *Gata5-Cre*-induced recombination occurred in mesenchyme the entire length of the OFT cushions (Figure 1A–C). Recombination occurred soon after mesenchyme cell formation or arrival (E11) in OFT and AV cushions (Figure 1D–Q), but did not start in the overlying endocardial cells until E12 (data not shown). We have previously shown that, in addition to endocardial cells, *Alk2* is strongly expressed in the newly formed AV cushion mesenchyme [26]. Similarly, *Alk2* is expressed in the OFT endocardium, as well as in the NC-derived and endocardium-derived OFT cushion mesenchyme (Figure 1R).

### Deletion of Alk2 in the post-EMT cushion mesenchyme leads to valve and septal defects

To investigate roles for Alk2 in mesenchyme during valve formation and maturation following EMT we deleted *Alk2* function there using the *Gata5-Cre* driver line. A role in these events for Alk2 expressed in other cell types in which *Gata5-Cre*-induced recombination occurs (epicardium and patches of differentiated myocardium) can be largely ignored as key epicardium-dependent events were not affected when *Alk2* function was deleted using the epicardial driver *Tbx18-Cre* (Figure S1) or in the working myocardium [26].

**Figure 2:**
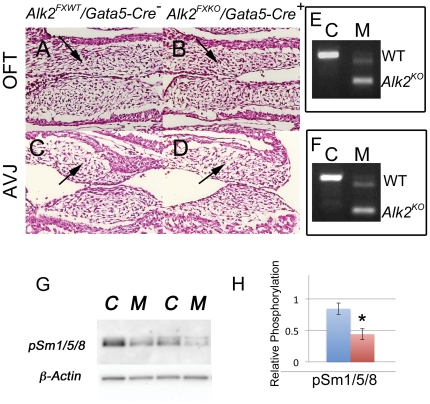
The overall cushion morphology is unaffected in *Alk2/Gata5-Cre* mutants at E11. H&E-stained sagittal sections through OFT cushions (A, B) and AV cushions (C, D) show plentiful mesenchymal cells in controls (A, C) and *Alk2/Gata5-Cre* mutants (B, D). Black arrows in A–D point to cushion mesenchyme. E–F, RT-PCR analysis of mRNAs from OFT (E) and AVJ (F) tissues at E11.5. C *Alk2^FXWT^/Gata5-Cre^−^* control; M *Alk2^FXKO^/Gata5-Cre^+^* mutant. The shorter amplification product (*Alk2^KO^*) predominant in mutant tissues confirms an efficient Cre-induced recombination. G, *Gata5-Cre*-induced recombination leads to reduction in Smad 1/5/8 phosphorylation in mutant (M) relative to control (C) E12.5 OFT tissue protein lysates analyzed by immunoblotting. H, Bar graph shows relative quantification of phospho-Smad1/5/8 in control (blue) and mutant (red) OFT tissues at E12.5 (normalized to β-actin; n = 3). Error bars, SEM; *p<0.05.

At E11, OFT cushions in *Alk2/Gata5-Cre* mutants contained numerous mesenchymal cells arranged in characteristic cushions indistinguishable from those of *Alk2/Gata5-Cre^−^* controls despite efficient recombination of the *Alk2* gene as shown by the RT-PCR assay using primers flanking exon 7 which is deleted in the recombined allele [32] (Figure 2), and reduced downstream signaling: a 50% reduction in Smad1,5,8 phosphorylation levels in OFT cushion tissues harvested at E12.

By E14, *Alk2/Gata5-Cre* mutants had developed abnormal cardiac phenotypes that showed high penetrance (Table 1). Seven out of nine mutant embryos displayed essentially bicuspid (two leaflet or bifoliate) aortic valves (BAVs) and/or perimembranous ventricular septal defects (VSDs) (Table 1). In all controls (*Alk2^FXWT^/Gata5-Cre^−^*) examined, fusion events to complete the ventricular septum had occurred and the aortic valves had three normal leaflets. No abnormal pulmonary valves, or defects in great artery development were found in controls or mutants.

**Table 1:** BAV and VSD in *Alk2/Gata5-Cre* mutants.

Stage	Genotype	Total	BAV (percentage)	VSD (percentage)
E14	Alk2^KOFX^/Gata5-Cre+	9	7 (78%)	7 (78%)
E17–E18	Alk2^KOFX^/Gata5-Cre+	6	5 (83%)	2 (33%)

**Figure 3:**
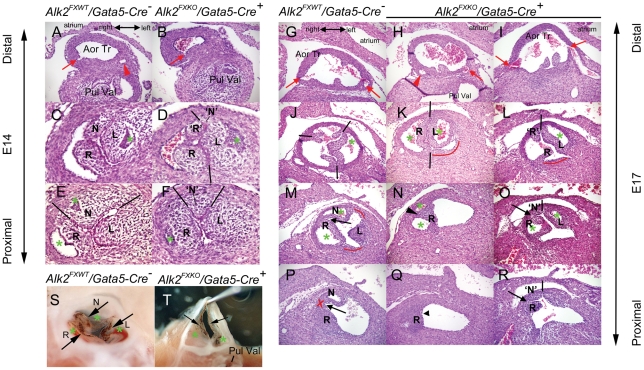
*Gata5-Cre*-induced deletion of *Alk2* function leads to defective development of aortic valves. **A–F**: Histological comparison of representative control (A, C, E) and mutant (B, D, F) aortic valves at E14; approximately transverse sections of the same valves at three levels. A, B: lower magnification at the level of coronary orifices (red arrow; coronary vessel: red arrowhead), where formed, distal to aortic valve leaflets showing normal development and relationship of aortic-side structures relative to pulmonary valve. All three control aortic leaflet cushions show evidence of excavation forming sinuses (green *): left (L) in panel C, right (R) and non-coronary (N) in panel E. N cushion occupies a similar segment of the circumference (black lines) as each of R and L (in E). In mutant valve, L (in D) and R (in F) leaflet cushions show normal excavation. Cushion ‘R’, although in the same position as N in control, is continuous with tissue R (panel D), not simply apposed as in control (in C). Nearer the base of the leaflet cushions (in E, F), mutant R is larger than control R. A small cushion (‘N’) is present between L and ‘R’ (in D, F), but shows no evidence of excavation and occupies a much smaller segment of the circumference (black lines in F) than N in control, so the valve is functionally bicuspid (bifoliate), with two excavating leaflet cushions, L and R/‘R’, apposing one another distally (in D). **G–R**: Histological comparison of representative control (A, J, M, P) and two functionally bicuspid mutant (H, K, N, Q and I, L, O, R) aortic valves at E17; approximately transverse sections of the same valves at four levels. G-I, at the level of coronary orifices showing their consistent position above right and left coronary leaflets, and consistent positioning of the leaflets in relation to adjacent tissues, in control and mutants. All three control aortic leaflet walls lie adjacent to well-developed sinuses (green *) left (L) in panel J, right (R) and non-coronary (N) in panel M. N leaflet occupies a similar segment of the circumference (black lines) as R and L (in J). Two examples of functionally bicuspid mutant valves are shown to illustrate key details of their morphology at this stage. The example in H, K, N, Q has features consistent with a fusion of R and N leaflets. Distally (K), only two leaflets, R and L, appose each other, with no small leaflet between them in the non-coronary position, each occupying about half the circumference (black lines). More proximally, the sinus of the R leaflet is divided into two (green *) by a raphe (black arrowhead) in N, but the leaflet does not then divide into two separate bases (arrowhead, Q). Distally the mutant valve shown in I, L, O, R also consists of two leaflets (in panel L; as R and ‘R’ are joined more proximally) that are not separated by a small cushion, but more proximally, one (‘N’) present. An area representing an interleaflet triangle (red curved line) can be identified between L and R leaflets in control (in M) and mutant (K, L) valves, and between L and N (control, in M) but the bases of the R and N leaflets even in control remain adjacent (in P, red X, black arrow) though not continuous (unlike Q). **S,T**: 6 month old adult control aortic valve with normal left (L) right (R) and non-coronary (N) leaflets (black arrows) and three sinuses (green*), and partially dissected functionally bicuspid aortic valve in *Alk2/Gata5-Cre* mutant, demonstrating two leaflets (black arrows) which appose only with each other across the entire lumen, and two sinuses (green *). Aor Tr, Aortic trunk; Pul Val, Pulmonary Valve.

Detailed histological examination (representative examples shown in Figure 3) were undertaken to define the morphology of the mutant aortic valves at E14 and E17, and to enable comparison with established rodent models of BAV [22] and bicuspid human aortic valves [33]. The abnormal valve phenotype was manifest as two large leaflet cushions and one much smaller, less developed, leaflet cushion in the non-coronary position at E14 (Figure 3F), and functionally bicuspid aortic valves (i.e. morphological evidence that the function of preventing backflow was being performed by only two, large apposing leaflets) at E17 (Figure 3K, L). By this later stage, a spectrum of abnormal right and non-coronary leaflet development was found, ranging from apparent fusion (as implied by the presence of a raphe, for example: Figure 3N) to the presence of a small, poorly developed cushion in the non-coronary position but slightly proximal to the level of apposition of the two, functional, leaflets (Figure 3O, R). Position and number of coronary orifices was normal in all mutants examined (Figure 3A, B, G–I) and these and the relative position of the pulmonary valve enabled the position of the abnormal/‘missing’ valve cushion to be consistently identified. Thus the bicuspid phenotype here more closely resembled the mouse BAV models in which *Gata5* or *Nos3* are deficient, as the hamster model involves common or fused right- and left-, and not non-coronary, leaflets. It was also notable that, even in E18 control valves, the right and non-coronary leaflet cushions had not yet become separated more proximally to form an ‘interleaflet triangle’, unlike the other leaflet pairs (Figure 3M,P). This maintained proximity may contribute to or reflect the mechanism by which this class of bicuspid valve persists into postnatal life.

45% of *Alk2/Gata5-Cre* mutants survived to 3 weeks postnatally (n = 53). In surviving postnatal mutants at two months and older, 3 of 22 examined had a functionally biscuspid aortic valve (Figure 3T) of the same pattern as identified prenatally. Overall, a spectrum of aortic valve phenotypes was present, including tricuspid, tricuspid with a smaller non-coronary leaflet, and functionally bicuspid with a very small third sinus and thickened non-coronary leaflet only revealed by sectioning (data not shown). Valves identified as functionally stenotic had irregularly thickened leaflets on sectioning whether bi or tri-cuspid (see the results below and data not shown).

**Figure 4:**
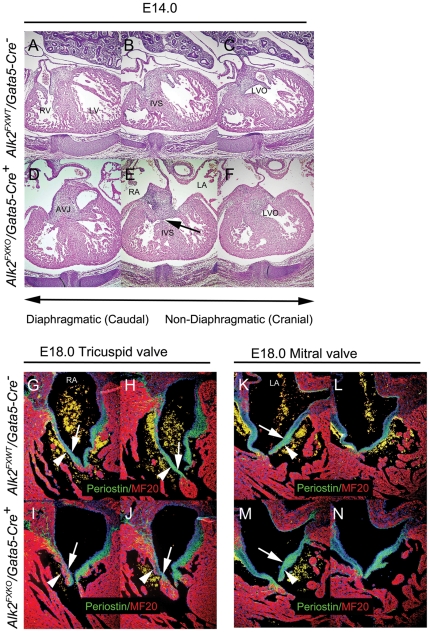
Ventricular septal defect and abnormal right atrioventricular valve leaflets in *Alk2/Gata5-Cre* mutants. H&E-stained sections of control (A–C) and *Alk2/Gata5-Cre* mutant (D–F) at three different levels showing perimembranous VSD in mutant (black arrow in E) but not control. Right (G–J) and left (K–N) atrioventricular valves from control (G, H, K, L) and *Alk2/Gata5-Cre* mutant (I, J, M, N) samples. Representative immunostained sections from two levels from each heart are shown: periostin (green), MF20 (red). Postero-inferior leaflet (G–J) shorter with less periostin-positive tissue (green) in mutant (I, J) than control (G, H) (arrows) and more myocardium (red: arrowheads). Control (K, L) and mutant (M, N) left AV valve leaflets do not differ (arrows, arrowheads). AVJ posterior side of atrioventricular junction; IVS muscular part of ventricular septum; LA, left atrium; LV, left ventricle; LVO, outlet part of LV leading to aortic valve; RA, right atrium; RV, right ventricle.

The VSD phenotype found in 7 of 9 mutant hearts at E14 was detectable in only 2 out of 6 mutants at E18, suggesting earlier VSDs likely represented a subtle delay in fusion that could still could be ‘rescued’ during the last four gestational days. Besides the aortic valves, *Alk2/Gata5-Cre* mutants also displayed abnormally short and muscular right atrioventricular valve leaflets at E18 when compared to corresponding control littermates, while the left atriocentricular valves appeared grossly normal (Figure 4).

### Cushion mesenchyme deficient in Alk2 showed reduced cell proliferation and altered gene expression

**Figure 5:**
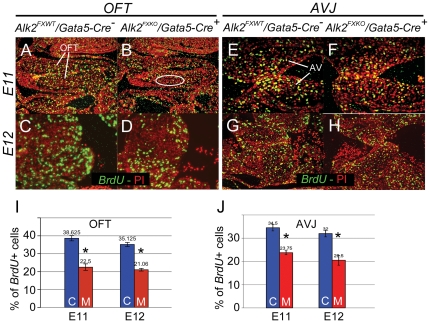
Less proliferation in endocardial cushion mesenchyme in *Alk2/Gata5-Cre* mutants. Representative images of anti-BrdU immunostaining (green-yellow) of control (A, C, E, G) and *Alk2/Gata5-Cre* mutant (B, D, F, H) sagittal sections at E11 (A, B, E, F) and E12 (C, D, G, H). A–D, OFT cushions; E–H, AV cushions. Mutant OFT cushion shows patchy incidence of BrdU- labeled nuclei (e.g., less in myocardium-adjacent region ringed by white ellipse). Bar graphs (I, J) illustrate differences in proportion of BrdU-labeled nuclei in the OFT (I) and AVJ (J) mesenchyme at E11 (left columns) and E12 (right columns) (n = 3) OFT, Outflow tract cushions; AV, atrioventricular cushions; C, control; M, mutant; Error bars, SEM; *p<0.05.

Maturation of cushion morphology is tightly coordinated with growth by cell proliferation and remodeling by programmed cell death. A 20–25% reduction in mesenchymal cell proliferation has previously been reported in *Gata5*-deficient BAV model mice [7], so we looked for differences in these processes between *Alk2/Gata5-Cre* mutants and controls. At E11 and E12, a period of rapid cushion growth, *Alk2/Gata5-Cre* mutant OFT cushions showed a 40% reduction in proliferation when compared to controls (Figure 5), but no detectable changes in apoptosis at E12 (data not shown).

**Figure 6:**
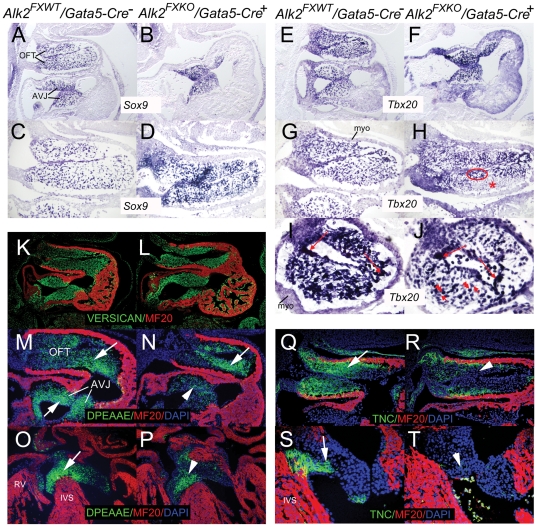
*Sox9* and *Tbx20* expression and less cleaved versican and tenascin-C in endocardial cushions and valve leaflets in controls and *Alk2/Gata5-Cre* mutants. In situ hybridization for *Sox9* (A–D) and *Tbx20* (E–J) on sagittal (A–H) and transverse (I, J) sections of E11 control and *Alk2/Gata5-Cre* mutant hearts. Prominent expression of both genes in atrioventricular (AVJ) and outflow tract (OFT) cushions in both control and mutant samples. Stronger staining detectable in certain regions of endocardium (*Tbx20*: red arrows in I, J) in equivalent areas of both control and mutant. Although staining for both *Sox9* and *Tbx20* in cushion mesenchyme also varied between adjacent cells (illustrated in J: short arrow lower vs double arrowhead higher), mutant but not control had groups of adjacent groups cells of lower (such as red asterisk in H) and higher (red ellipse) *Tbx20* signal. Immunostaining for total versican on sagittal E11 control (K) and *Alk2/Gata5-Cre* mutant (L) sections (green: versican; red: MF20). M–P, immunostaining for cleaved versican using anti-DPEAAE antibody on control (M,O) and *Alk2/Gata5-Cre* (N,P) sections. M–N, sagittal sections at E11; O–P, transverse sections at E14; green: cleaved versican; red: MF20. Staining in control AVJ mesenchyme (arrow in M,O) more extensive than mutant (arrowhead in N,P), though both similar in OFT. Immunostaining for tenascin-C on control (Q,S) and *Alk2/Gata5-Cre* mutant (R,T) sections; Q,R, sagittal sections at E11; S,T, transverse sections at E14. Green, tenascin-C (TNC); red, MF20. Staining in control mesenchyme (arrow) more extensive than mutant (arrowhead). AVJ, atrioventricular cushions; OFT, outflow tract cushions; IVS, ventricular septum; myo, OFT myocardium; RV, right ventricle.

Transcription factors and ECM components have been proposed to contribute to cushion morphogenesis. Sox9 and Tbx20 have been reported as positive regulators of cushion mesenchyme proliferation and Bmp2, a possible ligand for Alk2, shown to induce *Tbx20* expression indirectly in vitro [23], [24], [25] so we examined their expression by in situ hybridization (ISH). There was no difference in staining pattern in cushion endocardium for either RNA between control and mutant, including areas of higher expression (Figure 6I,J). In cushion mesenchymal cells, expression of both genes was easily detected in both control and mutant cushions (Figure 6A–J) suggesting no simple dependence on Alk2 function. A more detailed examination of the relative stain intensities within the same section showed variation amongst adjacent mesenchyme cells in the same cushion. While some of this may be attributable to cells cut by sectioning there are areas where all the cells appear less stained (for *Tbx20* in mutant OFT cushion: Figure 6H; the same regional pattern was obtained on a sister section (data not shown).

**Figure 7:**
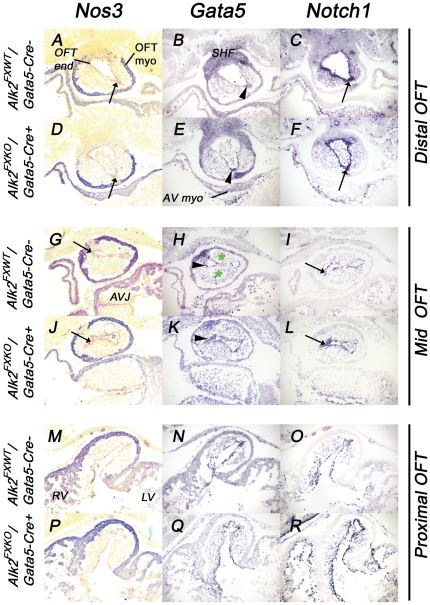
Regional patterns of BAV-associated gene expression do not differ between control and *Alk2/Gata5-Cre* mutant outflow tracts. Sections transverse to distal (A–F), mid (G–L) and proximal (M–R) outflow tracts of control (A–C; G–I, M–O) and mutant (D–F; J–L; P–R) embryos showing expression patterns of Nos3 (Immunostaining: Nos3, pink; myocardium, blue-purple; nuclei, yellow; images color-inverted so that signal in single layer of endocardium is visible), (A, D, G, J, M, P); *Gata5* (ISH: B, E, H, K, N, Q) and *Notch1* (ISH: C, F, I, L, O, R). Staining for Nos3 and *Notch1* present in virtually all endocardial cells in the OFT; *Gata5* (e.g., arrow heads) detected above background in endocardium lateral to rather than over the two main cushions (septal, parietal: *) where *Notch1* and Nos3 also more strongly expressed (arrows). Non-coronary leaflet develops from the intercalated cushion on the left in these images, right coronary from the adjacent part of the parietal cushion (* in the more anterior position). *Gata5* also detected in second heart field (SHF) cells/most distal OFT wall. OFT, myo: outflow tract myocardium; OFT end, OFT endocardium; AV myo, atrioventricular myocardium; AVJ, atrioventricular cushion; RV, right ventricle; LV, left ventricle.

It was recently shown that proteolytic cleavage of versican, a large extracellular matrix proteoglycan, is an important step in the regulation of late embryonic valve development and that a failure in this process leads to formation of thick myxomatous valves [34]. While versican protein levels were comparable between *Alk2/Gata5-Cre* mutants and controls (Figure 6K–L), immunostaining also detected less cleaved versican in mutant AV, but not OFT, cushions (Figure 7). In addition, immunostaining for the extra-cellular matrix glycoprotein, tenascin-C, expression of which has been shown to be associated with areas undergoing major structural changes during OFT morphogenesis in the chick [35], was reduced in both OFT cushions and in developing AV valves (Figure 6Q–T). These results imply that Alk2-mediated signaling is involved in regulation of extracellular matrix deposition and/or remodeling. However, the lack of differences in anti-DPEAAE-positive immunostaining between *Alk2* mutant and control OFT cushions argues that Adamts-induced versican cleavage here is not involved in development of the aortic valve phenotype observed in *Alk2/Gata5-Cre* mutants.

Despite a reduced proliferation rate in OFT mesenchyme at E11 and 12, sufficient mesenchymal cells were present in time for both AV and OFT septation to occur in mutants, However, other cushion-related abnormalities occurred subsequently, so subtle defects in expression and processing of molecules such as those discussed above might still leave *Alk2/Gata5-Cre* mutants more vulnerable to morphological abnormalities and environmental or epigenetic effects.

### Expression of key regulators associated with BAVs or valve maturation

It has previously been shown that defective function of *Notch1, Gata5* or *Nos3* leads to BAV development, and that, where investigated, the abnormal morphology was already detectable as early as E11.5 [7]. To examine whether abnormal expression of these genes was part of the mechanism underlying our model we therefore compared the mRNA expression patterns of using ISH (*Notch1*, *Gata5*) or immunofluorescence (Nos3) at E11, and at several levels of the OFT to ensure that regional defects in expression, perhaps associated specifically with the future intercalated cushion development, were not missed. In both control and mutant sections, the expression of all three genes was detected in AV and OFT endocardium, and in a somewhat similar, non-uniform, way both along the length of the OFT and around its lumen (Figure 7). *Notch1* and Nos3 were detected in most if not all OFT endocardial cells but staining was stronger (or appeared sooner) more distally, and in lateral areas between adjacent cushions and where the intercalated cushions were developing. *Gata5* endocardial expression was harder to detect but present in endocardium adjacent to rather than over the two main cushions at distal and mid-levels, the same areas in which *Notch1* and Nos3 staining was also higher (Figure 7G–L). They were all detected in endocardium over the proximal cushion as it extends into the ventricle (Figure 7M–R). We also examined expression of *Notch1* target genes *Hey1* and *Hey2* (Figure S2). In OFT endocardial cells, *Hey1* expression was highly restricted, principally to the endocardium between the main cushions, similar to that of *Gata5* (see above). *Hey2* expression was detected in most OFT endocardial cells, in a pattern very similar to that of *Notch1*. In AV endocardium, expression of both these genes appeared in only a small subset of *Notch1*-positive cells, *Hey1* being almost completely undetected. Again we could demonstrate no consistent differences in expression between control and mutant tissues.

Levels of mesenchymal staining present in sections probed for these genes were too low to interpret and compare (Figure 7, Figure S2 and data not shown). Nos3 expression was not detected above background in mesenchyme cells by immunofluorescence. In non-endocardial cells, *Gata5* and *Hey1* expressions were detected in overlapping domains of second heart field cells of the distal OFT, *Hey1* expression in atrial appendage myocardium and a regional population of epicardial cells, *Hey2* in ventricular wall myocardium and Gata5 weakly in AV and some OFT myocardium (Figure 7, Figure S2 and data not shown).

From this detailed examination of expression of genes previously associated with BAV, we conclude that although there were regional differences in gene expression within the OFT consistent with a role for these genes in the development of the intercalated cushions, there were no differences in these patterns between control and mutant samples. Despite the similarity of bicuspid phenotype (R-N fusion) with that reported in *Gata5* and *Nos3* mutants, these results provide no evidence that Alk2-mediated signaling leads to mis-expression of genes known to be associated with BAV, suggesting that the pathogenetic mechanism leading to BAV in these Alk2 mutants is novel or acting downstream of these genes.

### Functional defects in adult Alk2/Gata5-Cre mutants

**Figure 8:**
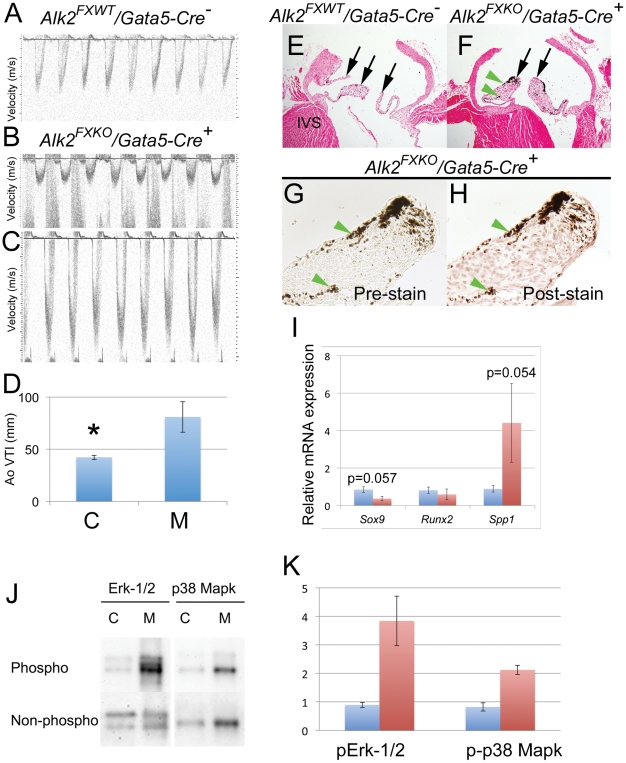
Aortic stenosis and insufficiency in *Alk2/Gata5-Cre* mutants. Echocardiography of a control (A), an *Alk2/Gata5-Cre* mutant with aortic stenosis and insufficiency (B) and an *Alk2/Gata5-Cre* mutant with aortic stenosis (C). A bar graph (D) depicts the two-fold difference in aortic velocity index between controls (C) and stenotic mutants (M) (n = 6). Aortic valve leaflets (arrows) of stenotic *Alk2/Gata5-Cre* mutant thickened and hypercellular (F) when compared to those of age- and sex-matched control (E) (Von Kossa-stained sections with nuclear red counterstain). Dark patches are endogenous melanin, not evidence of calcification: Alizarin Red staining on sister section to (F) (pre-staining, G; post staining H) shows dark patches (e.g., green arrowheads) match precisely and no bright red positive stain for calcium. I, Relative real-time RT-PCR quantification of *Sox9, Runx2* and *Spp1 (osteopontin)* in isolated valve leaflets of controls and *Alk2/Gata5-Cre* mutants with aortic stenosis and insufficiency (n = 2). J, Up-regulation of phospho-Erk1/2 and phospho–p38 in samples with aortic stenosis and insufficiency (n = 2). Bar graph (K) depicts relative quantification of phospho-Erk1/2 and phospho-p38 between controls and *Alk2/Gata5-Cre* mutants shown in J (n = 2). Error bars, SEM; *p<0.05.

As mentioned above, 45% of mutant mice survived to adulthood. We analyzed aortic valve function in 22 of these mice at 3–18 months using echocardiography (Figure 8A–C). Hemodynamic evaluation of *Alk2/Gata5-Cre* mutant and age/sex-matched control mice (see methods) demonstrated an increased velocity gradient across the aortic valve of 6 mutant mice consistent with aortic stenosis (Figure 8D). In addition, two of the six mice with stenosis displayed regurgitation indicative of aortic insufficiency (Figure 8B). Subsequently, the mice were euthanized and aortic valve tissues further analyzed grossly, then histologically (as also reported above) or for gene expression by real-time quantitative RT-PCR (below). Histological analyses showed that mutant valve leaflets were irregularly thickened and hypercellular when compared to corresponding controls (Figure 8E–F) typical of stenotic valve leaflets, though elevated cell proliferation was not detected (Figure S3B). Such features might be expected at least to compromise flexibility, resulting in stenosis, as well as efficient apposition, resulting in insufficiency.

We harvested aortic valve tissue from three functionally abnormal *Alk2* mutants and three functionally normal age/sex-matched controls and analyzed them for expression of selected genes associated with osteogenic differentiation or inflammation implicated in human valve disease [36]. The results imply that inflammatory pathways were not involved in the pathogenesis as there were no genotype-consistent changes in *Alox5 or Cd68* expression between mutant and control tissues (Figure S3). In contrast, mutant tissues showed a clear tendency for higher *osteopontin* (*Spp1, p = 0.054*) and lower *Sox9* (p = 0.057) expression when compared to corresponding control tissues (Figure 8I). Moreover, the *Spp1* and *Sox9* expression levels were inversely related to one another (Figure S3A), consistent with the recent model proposed by Peacock et al [37]. Since reduced levels of *Sox9* have been shown to be causally related to valve ossification [37], the observed differences are consistent with a model in which affected valve tissues in *Alk2* mutants with aortic stenosis and insufficiency display pro-osteogenic changes. However, expression of the key osteogenic transcription factor *Runx2* was consistently lower in mutant valve tissues than in controls, and valve calcification was not detected by von Kossa or Alizarin Red staining (Figure 8G–H). We also analyzed activation of Bmp Smads (Smads1/5/8), Erk1/2 and p38 Mapk using Western blotting and probing with phospho-specific antibodies. Phosphorylation levels of Bmp Smads were very low and did not show detectable differences between mutants and controls. In contrast, the samples harvested from *Alk2* mutants that showed both stenosis and insufficiency showed consistent, robust increase in Erk1/2 phosphorylation, and a less pronounced increase in p38-Mapk phosphorylation (Figure 8J–K). Prolonged Erk1/2 activation has been shown to lead to calcification in valve interstitial cell cultures *in vitro* [38]. Although the limited sample number (n = 2) does not allow us to establish statistical significance, these experiments suggest that the increase in Erk1/2 phosphorylation we observe contributes to pro-osteogenic expression pattern shown in Figure 8I.

## Discussion

Abnormal *Alk2* function has been implicated in human cardiac disease [29], [30]. However, our current knowledge about the role of *Alk2* in cardiac development is limited to endocardial EMT and neural crest cell migration [26], [39], [40], and it is not known which Alk2-mediated processes fail in human patients that show disease-causing mutations in the *Alk2* gene. Since EMT fails in *Alk2/Tie2-Cre* mutants, efficient analysis of a later role for Alk2 in endocardium or its derivatives in post-EMT septum and valve development in isolation is not possible using this model. Similarly, in *Alk2/Wnt1-Cre* mutants, recombination in neural crest cells occurs very early and affects not only the initial distribution of these cells in the OFT but also causes malformations in adjacent structures that could affect valve morphogenesis indirectly [39]. To overcome these limitations, we used the *Gata5-Cre* transgenic mouse line. This was originally designed as an epicardium-specific driver but this and our earlier studies have shown that *Alk2* does not play a detectable essential role in the epicardium (Figure S1) or working myocardium [26]. Recombination in endocardium commences after the main phase of Alk2-dependent EMT, and, in contrast with AV events, no defects in OFT septation or arterial valve development were detected in *Alk2/Tie2-Cre* mutants [26]. In the present study we show that *Gata5-Cre* induces efficient recombination in all cushion mesenchyme soon after EMT or migration. Using this driver line, we found that, following abrogation of *Alk2* in the endocardial cushion mesenchyme, cushion growth was attenuated, and VSD and functional BAVs developed.

In a recent detailed analysis of BAV development [11], two morphologically and etiologically different rodent models were studied: the mouse model lacking *Nos3*, where BAVs results from right and non-coronary (R-N) leaflet precursor fusion present at or before OFT septation occurs, and a well-established hamster model where right-left coronary (R-L) leaflet fusions are associated with abnormal OFT septation morphology. The R-N and R-L BAV phenotypes are the two more common in humans, and have different clinical implications. In pediatric patients, R-N BAVs are more likely to progress rapidly to aortic insufficiency and stenosis, while those with R-L BAVs develop more rapid aortic wall regeneration [3]. Our analysis of the *Alk2/Gata5-Cre* mutant aortic valve during its embryonic morphogenesis shows no evidence of BAV formed by R-L leaflet fusion, but a spectrum of defects including fusion of R-N leaflet precursors, and functional bicuspid valve composed of separate right and left leaflets each adjacent to a coronary orifice, and only a very small, poorly formed leaflet in the non-coronary position. Genetic mutants surviving to adulthood were more likely to develop stenosis and insufficiency than controls. In investigating the mechanism underlying these phenotypes, we looked for a relationship with the *Nos3* (and related *Gata5*) R-N fusion models. We could find no difference in expression of *Nos3* or *Gata5* immediately prior to the stage at which morphological abnormalities were apparent in these models, suggesting that Alk2 lies downstream or a different pathway is involved. Although the category of BAV resulting from altered expression of Notch1 signaling has not been reported, we also established that expression of *Notch1* itself was not altered prior to cushion maturation. Notch signaling is also implicated in preventing calcification, which is frequently associated with thickened or mechanically compromised valves with stenosis in humans, but no evidence of calcification was found in our adult mutant mice with stenosis suggesting that either Notch signaling is not compromised, or other aspects of the abnormal phenotype of the valve cells were simultaneously protective. Some pro-osteogenic differences (lower *Sox9* and higher *Spp1* expression and higher Erk1/2 phosphorylation in mutant valve leaflets) were detected, but not others: *Runx2* expression and Bmp Smad phosphorylation were not elevated. Calcification in mouse valves with compromised Notch1 signaling results only after a further perturbation (high cholesterol diet) [41] and involves inflammatory cells in which abnormal Notch signaling is also implicated as responsible for their contributing to the calcified phenotype as well as valve interstitial cells. We found no evidence of higher expression of two markers of inflammation in stenotic adult *Alk2/Gata5-Cre* mutant aortic valves, suggesting that inflammation was not causal to the stenosis. Together with absence of calcification, reduced Notch signaling as a result of *Alk2* deficiency in the valves of adult *Gata5-Cre/Alk2* mutants valves is also unlikely.

Further to establishing the mechanism underlying the BAV phenotype present by E14, we examined other aspects of cell behavior in the precursor tissues. *Alk2/Gata5-Cre* mutant cushions contained plenty of mesenchymal cells, and cushion organization closely resembled that in controls soon after recombination. However, we detected a 40% reduction in mesenchymal cell proliferation rate at E11 and E12, greater than the 20–25% reported for *Gata5*-deficient cushion mesenchyme [7], and reduced phosphorylation of Bmp Smads in OFT tissues at E12. Transcription factors Sox9 and Tbx20 have been implicated in promoting proliferation over differentiation in cushion mesenchyme and we have found some evidence that our mutant cushions contain patches of mesenchyme with reduced *Tbx20* expression. Some altered expression of cleaved versican and tenascin-C were also found, but there was no simple correlation with later morphological differences between control and mutants. Levels of cleaved versican were reduced in AV cushions but an increase in cellularity was not observed. Subtle abnormalities in *Alk2/Gata5-Cre* right atrioventicular but not left atrioventricular AV valve leaflets were detected at E18 but the tenascin-C expression adjacent to both valves was altered in mutants. AV and OFT septation, which might be expected to be compromised by a reduction in mesenchyme cell number, still occurred, and the incidence of VSDs was reduced by more than 50% between E14 and E18, suggesting that abnormalities created by Alk2 signaling deficiency might be rescued or ameliorated over time.

Aortic leaflets of *Alk2/Gata5-Cre* mutants did not appear of abnormal thickness or cellularity at a late embryonic stage. Leaflets from mutant adult valves with stenosis were abnormally thickened, in common with diseased human valves (data not shown), but we did not detect evidence of higher cell proliferation than that in control valve tissues after diagnosis, so the timing and mechanisms underlying this process remain to be determined, and may be secondary to the morphology rather than the altered Alk2 function. Even in aortic valves with three leaflets, there is a correlation between developing valve disease and the deviation of leaflet size away from equality in humans. The abnormal mechanical stresses on valve leaflets in these settings could lead to abnormal organization and repair of valve components [18], even without the direct effects of an abnormal valve cell genotype being taken into account.

Detailed studies by others [7], [11] have started to provide information about the morphological events underlying the development of BAV in rodents during embryonic development. Our work here on the *Alk2/Gata5-Cre* model also reveals a novel morphological feature not reported by others. Rather than only two leaflet cushions, or a simple R-N, or fusing intermediate phenotype, being present by E14, and at E17, our evidence demonstrates that in most cases a small third cushion in the non-coronary position was still present in mutant aortic valves, although the other two leaflets appose one another across the lumen and are performing the valvar function at this time. The presence of such a structure may further compromise function after birth if it maintains its relative size by impeding apposition of the other valve leaflets. It remains to be determined why this structure and not the pulmonary equivalent, is altered. Both are formed from intercalated cushions in the OFT, and, as our detailed ISH analysis here shows for the first time, expression of BAV-implicated genes *Nos3, Gata5, Hey1 and Notch1* was highest in the OFT endocardium in ‘lateral’ positions that include where the intercalated cushions develop immediately before morphological abnormalities are first detectable in these models. All OFT cushions contain mesenchymal cells formed by EMT, but, unlike the larger (parietal and septal) cushions, intercalated cushion mesenchyme includes a molecularly distinct population that may be of pharyngeal mesoderm origin (Robert Anderson, personal communication (Sizarov et al 2012 in press)), as well as far fewer neural crest cells [11]. However, as the intercalated cushions lie on opposite sides of the OFT lumen, they are also adjacent to myocardial populations that differ in their molecular identity [42], and, as the aortic and pulmonary valve precursors are differently positioned within the OFT relative to other structures, they may experience different flow conditions even before septation [43]. Experimentally altered flow patterns can result in abnormal cushion formation in earlier embryos [44] and in valve leaflets [45] and secondary shear stress or flow-related mechanisms might contribute to the phenotypes identified in animal models of BAV. *Nos3* expression is regulated by flow [46], [47], which affects endothelial motility and EMT [48]. Moreover, Fernandez et al [21] suggest a mechanism whereby *Nos3* deficiency results in abnormalities in endocardial generation of mesenchyme (EMT), which leads to a failure to form separate precursor cushions for the right and non-coronary leaflets. Notch1 signaling is also implicated in EMT and this might contribute to its role in BAV in humans. It is important to note that in none of the descriptions of animal BAV models [7], [49](this paper) is there definitive evidence of fusion between separate, fully formed, excavated leaflets, and that therefore the term ‘fused’ or ‘fusion’ used when defining BAV morphology should not be presumed to imply such a process. Instead the BAVs in both *Nos3* (and perhaps *Gata5*) –deficient R-N mice and R-L hamster phenotypes originate as a lack of failure to develop separate leaflets through the failure to develop normal separate leaflet cushions at a much earlier stage. Nevertheless, further remodeling of mature valve leaflets through flow-mediated or other mechanisms, and early death, might be expected to contribute to the variable penetrance of BAV and other phenotypes in postnatal animals in this and other models (Nos3^−/−^: 5/12 BAV in adults [6]; Gata5-/- 25% BAV in adults, 3% in controls [7]; Nkx2-5+/−: 0%, 2%, 11% depending on background, up to 8 times that of controls [5]). Inadequate function of the more abnormal aortic valves at birth, and reduced cardiac efficiency where ventricular septation had not occurred are obvious candidates to account for the 55% death rate amongst early postnatal *Alk2/Gata5-Cre* mutants and thus reduce the penetrance of BAV greatly from its late embryonic rate.

The absence of OFT septal defects and the reduction in penetrance of VSD during prenatal development ventricular septation are consistent with roles for *Alk2* in septal and valvar maturation that are partially redundant or subject to genetic modifiers that affect the severity of the phenotype, or involve processes in which there is a degree of plasticity in timing. Whether always three leaflet, or the result of postnatal ‘recovery’ from a prenatal functional BAV phenotype, the incidence of stenosis argues that three leaflet aortic valves in *Alk2* mutants still remain susceptible to stenosis and insufficiency.

## Materials and Methods

### Mice

Mice carrying the conditional *Alk2-flox* allele (*Alk2^FX^*) and *Gata5-Cre* transgenic mice were used and genotyped as previously described [31], [39]. *Tbx18-Cre* mice were kindly provided by Dr. S Evans [50] and were genotyped for the presence of *Cre* by PCR (oligo sequences; Cre-F, 5′-cgttttctgagcatacctgga-3′; Cre-R, 5′-attctcccaccgtcagtacg-3′). Timed matings between male mice that were heterozygous for the *Alk2* knockout allele (*Alk2^WTKO^*) carrying a heterozygous *Gata5-Cre* (or *Tbx18-Cre*) transgene, and female mice homozygous for the *Alk2-flox* allele (*Alk2^FXFX^*) were used to obtain *Alk2^FXKO^/Gata5-Cre^+^* tissue-specific mutant embryos (herein called *Alk2/Gata5-Cre*). As controls, we used *Alk2^FXWT^/Gata5-Cre^−^* littermates, which have two functional *Alk2* alleles.

### Ethics Statement

This study was carried out in strict accordance with the recommendations in the Guide for the Care and Use of Laboratory Animals of the National Institutes of Health. The experiments described in this study were specifically approved by the University Committee on Use and Care of Animals of the University of Michigan-Ann Arbor (Protocol Number: #09944).

### *R26R* lineage tracing

For lineage tracing experiments, timed matings were set up between *Alk2^WTKO^/Gata5-Cre^+^* males and *Alk2^FXFX^/R26R^lacz/lacz^* females as previously described [51]. The embryos were harvested (E10-E11) and stained for the presence *of β-galactosidase* activity as described [52].

### Histology, immunohistochemistry, apoptosis and BrdU incorporation assays

Embryos were fixed in 4% paraformaldehyde for 12–18 hours at 4°C, dehydrated and embedded in Leica Histowax according to standard procedures. After embedding, sections were cut at 5–7 µm. Histological analysis was performed on sections stained with hematoxylin and eosin according to standard procedures. For immunohistochemistry, citrate antigen retrieval was performed where necessary (and before anti-BrdU antibody use, where combined staining was performed). Primary antibodies (listed below) were detected using either Alexafluor-594 or Alexafluor-488 secondary antibodies (Invitrogen) on sections mounted in Vectashield containing DAPI or propidium iodide nuclear counterstains (Vector Labs Inc). Primary antibodies to the following used were: periostin (Abcam ab14041), versican (Chemicon AB1033), cleaved versican (DPEAAE) (Thermoscientific PAI-86354), MF20 (DSHB), Nos3 (eNos) (Thermoscientific RB9279-P) and WT1(Santa Cruz Biotechnology sc-192). Apoptotic cells were detected using the ‘Dead End’ TUNEL assay kit (Promega) following manufacturer's instructions. For cell proliferation analyses i.p. injections of cell proliferation labeling reagent (RPN201, Amersham/GE Healthcare) were performed. BrdU-labelled cells were detected on sectioned wax-embedded tissues using anti-BrdU antibody (RPN202, Amersham/GE Healthcare) according to manufacturer's instructions. Fluorescent images were viewed on an Olympus BX51 microscope and documented using an Olympus DP71 camera. Adobe Photoshop CS4 was used to invert the image colors to enable the results to be visible. Each assay was repeated at least once; see figure texts for details.

### In situ hybridization (ISH)

For RNA in situ hybridization, embryos were fixed in 4% buffered paraformaldehyde for 12–18 hours, dehydrated and either stored in 95% ethanol at −20°C until needed or processed for embedding in Leica Histowax or BlueRibbon embedding medium. In situ hybridization was performed [53] on 10 µm sections using non radio-active DIG-labeled RNA probes made according to manufacturer's instructions (Roche Applied Science). Each analysis was performed on 2–3 independent samples per genotype. Probes for *Sox9, Tbx20, Notch1* and *Hey2* were made from templates kindly provided by Nobby Kamiya [54], Richard Harvey [55] and Irma Thesleff [56], respectively. Probes for *Alk2* (nts 814–1414 and nts1281–2024 of NM_001110204) *Gata5* (nts 1460–2061 of NM_008093) and *Hey1* (nts 205–1105 of NM_010423) were made from cloned templates containing PCR-amplified sequences from total mouse cDNA. Results were viewed on an Olympus BX51 or Leica MZ95 microscope and documented using an Olympus DP71 camera and software.

### Real-time Quantitative PCR

Total RNAs were isolated from individual aortic valves (trimmed to include mainly leaflets) placed in RLT buffer (Qiagen) and DTT using the RNeasy microkit (Qiagen), and used immediately to synthesize cDNA using Omniscript reverse-transcriptase (Qiagen) and random priming according to manufacturer's instructions. cDNA aliquots (one tenth each total cDNA prep) were then pre-amplified for 10 cycles using Taqman PreAmp Mastermix (Applied Biosystems) and the same batch of diluted mix of primers used for subsequent quantitative PCR assays, and the resulting products diluted, all according to manufacturer's instructions. β-*actin* (*Actb*) primers were included in the pre-amplification mix. Real-time qPCR analyses were performed using Universal ProbeLibrary-based assays (Roche Applied Science) with gene-specific primer sequences generated by the manufacturer's online site (https://www.roche-applied-science.com/sis/rtpcr/upl/index.jsp?id=UP030000). Each 30 µl reaction included 2 µl of sample as prepared above, 1 µl each forward and reverse primer (stock 20 µM), 0.5 µl Probe and 15 µl Taqman Universal PCR Master Mix (Applied Biosystems). 45-cycle assays were performed using the ABI 7300 PCR and Detection System (Applied Biosystems) and analysed using 7500 System v1.2.2 software. β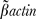 was used to standardize relative quantities. Results with C_t_s outside 15–30 cycles were excluded. cDNAs from aortic valve tissue from three different control and mutant mice were analyzed simultaneously.

### Immunoblotting

For Western blots, tissues were lysed in 2× Laemmli sample buffer [57], heated at 80°C for 10 minutes, and quantified by Quant-It protein assay reagents (Invitrogen). Samples (3 µg protein per lane) were loaded onto NuPaGE 4–12% Bis-Tris gels (Invitrogen) and run at 200 V for 50 minutes. Proteins were transferred onto nitrocellulose filters using an iBlot dry blotting system (Invitrogen). For immunodetection, filters were blocked with 5% milk for 1 hour and incubated with primary antibodies for 12–16 hours at +4°C in 2%milk. Primary antibodies to the following antigens were used (all Cell Signaling Technology unless otherwise stated) phospho-Smad 1,5,8 (9511), phospho-Erk1/2 (4376), Erk1/2 (4695), phospho-p38Mapk (4511), p38Mapk (Santa Cruz Biotechnology sc-728), *β-actin* (Sigma A1978). The filters were washed in TBST, incubated for 30 mins with HRP-labeled secondary antibodies washed again in TBST and incubated for 5 minutes in Immobilon HRP Substrate (Millipore). Detection and quantification was accomplished by using the BioSpectrum AC imaging system (UVP).

### Echocardiography

Induction of anesthesia was performed in an enclosed container filled with 4% isoflurane. After induction, the mice were placed on a warming pad to maintain body temperature. 1–1.5% isoflurane was supplied via a nose cone to maintain a surgical plane of anesthesia. The hair was removed from the upper abdominal and thoracic area with depilatory cream. ECG was monitored via non-invasive resting ECG electrodes. Transthoracic echocardiography was performed in the supine or left lateral position. Two-dimensional, M-mode, Doppler and tissue Doppler echocardiographic images were recorded using a Visual Sonics' Vevo 770 high resolution *in vivo* micro-imaging system. LV ejection fraction was measured from the two-dimensional long axis view. Systolic and diastolic dimensions and wall thickness were measured by M-mode in the parasternal short axis view at the level of the papillary muscles. Fractional shortening and ejection fraction were also calculated from the M-mode parasternal short axis view. Diastolic function was assessed by conventional pulsed-wave spectral Doppler analysis of mitral valve inflow patterns (early [E] and late [A] filling waves). Doppler tissue imaging (DTI) was used to measure the early (Ea) diastolic tissue velocities of the septal and lateral annuluses of the mitral valve in the apical 4-chamber view. Echo was performed on 3 month-old (3 male, 3 female), 7.5 month-old (4 male, 4 female) and 13 month or older (4 male, 4 female) *Alk2/Gata5-Cre* mutants, along with male and female control animals of the same ages.

### Histological detection of calcification

Von Kossa staining with nuclear red counterstaining was performed on rehydrated 7 µm formaldehyde-fixed tissue sections using the Diagnostic Biosystems kit (Pleasanton CA) according to manufacturer's instructions. Alzarin Red staining was performed on sister sections with freshly prepared alizarin red using a standard protocol. Resulting sections were viewed on an Olympus BX51 microscope and documented using an Olympus DP71 camera and software.

### Statistical analysis

Statistical analysis was done by Mann-Whitney U test. P values<0.05 were considered significant. Error bars represent SEM in all bar graphs.

## Supporting Information

Figure S1 **No detectable cardiac defects in epicardium-specific** ***Alk2*** **mutants.** Coronary vasculature smooth muscle and cardiac fibroblast cells are both epicardium-derived. R26R-driven βgal staining (blue) shows that abrogation of *Alk2* function in epicardial cells using the *Tbx18-Cre* driver line did not cause detectable defects in the smooth muscle cell layer surrounding coronary arteries (E18: white arrows in A, B) or in generation and migration of epicardially derived WT1-positive cells (white arrowheads) into the ventricular walls (C, D) (E13: immunostaining for WT1, green; for MF20, red). A and C, controls; B and D, *Alk2/Gata5-Cre* mutants.
(TIF)

Figure S2 **No difference in regional patterns of** ***Notch1*** **target genes** ***Hey1*** **and** ***Hey2*** **between control and** ***Alk2/Gata5-Cre*** **mutant outflow tracts.** ISH performed on sections transverse to distal (A–D), mid (E–H) and proximal (I–L) outflow tract of control (A, B, E, F, I, J) and mutant (C, D, G, H, K, L) embryos showing expression patterns (blue) of *Hey1* (A, C, E, G, I, K) and *Hey2* (ISH: B, D, F, H, J, L). *Hey1* expression in OFT endocardium (arrow head) restricted to cells lateral to but not over the two main OFT cushions (parietal, septal *). ISH staining for *Hey1* on sagittal sections (distal to the left) of control (I′) and mutant (K′) also not above background in mesenchymal cells of the proximal OFT cushions (Prox OFT). *Hey2* detected in most OFT endocardial cells but stronger in areas lateral to the main OFT cushions (arrows). Non-coronary leaflet will develop from the intercalated cushion on the left side in these images. *Hey1* also detected in atrial appendage myocardium (A Myo) and epicardium (green arrow). *Hey2* also detected in ventricular wall myocardium (V Myo).
(TIF)

Figure S3 **Expression findings in valve leaflets of** ***Alk2/Gata5-Cre*** **mutants with aortic stenosis and insufficiency.** A, Scatter plot demonstrates inverse relationship between *Spp1* and *Sox9* expression in aortic valve leaflets of stenotic *Alk2/Gata5-Cre* mutants measured by real-time RT-PCR. B, Bar graph illustrates no difference in relative expression of inflammation markers *Alox5* and *Cd68*, and of proliferation marker *Pcna*, in stenotic mutant (red) and normal control (blue) aortic valve leaflets measured by real-time RT-PCR quantification (n = 3). Error bars, SEM.
(TIF)
