# Supplementary figures and images for: Deficient Signaling via Alk2 (Acvr1) Leads to Bicuspid Aortic Valve Development
Source: PLoS One. 2012 Apr 19;7(4):e35539. doi: 10.1371/journal.pone.0035539 (PMC3334911; doi:10.1371/journal.pone.0035539)

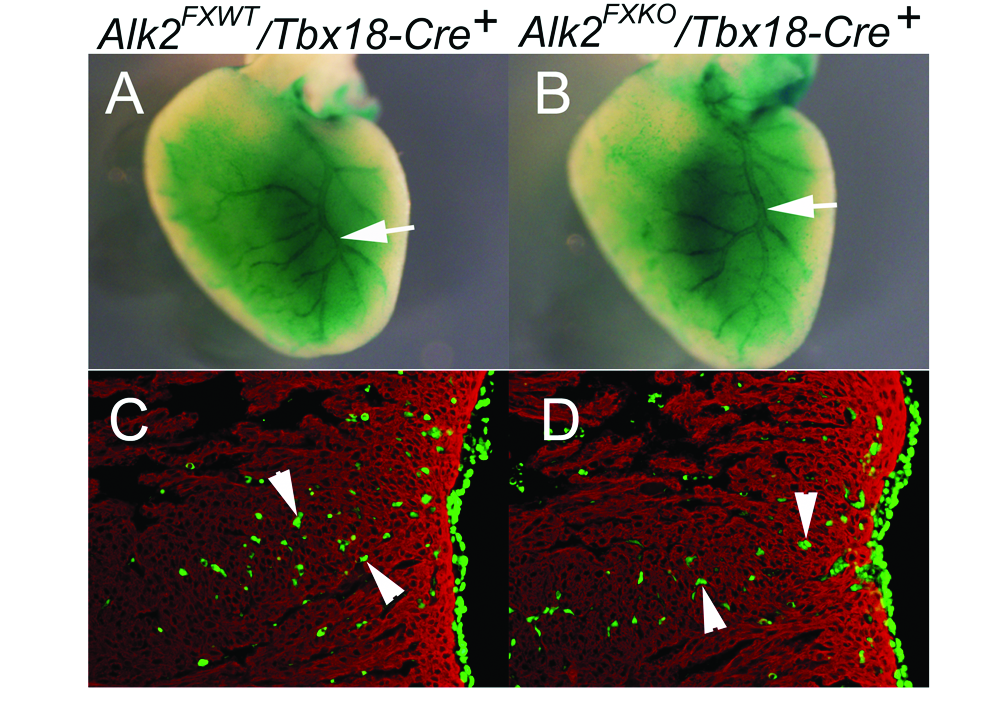

Supplement: Figure S1 — No detectable cardiac defects in epicardium-specific Alk2 mutants. Coronary vasculature smooth muscle and cardiac fibroblast cells are both epicardium-derived. R26R-driven βgal staining (blue) shows that abrogation of Alk2 function in epicardial cells using the Tbx18-Cre driver line did not cause detectable defects in the smooth muscle cell layer surrounding coronary arteries (E18: white arrows in A, B) or in generation and migration of epicardially derived WT1-positive cells (white arrowheads) into the ventricular walls (C, D) (E13: immunostaining for WT1, green; for MF20, red). A and C, controls; B and D, Alk2/Gata5-Cre mutants. (TIF) [file pone.0035539.s001.tif]

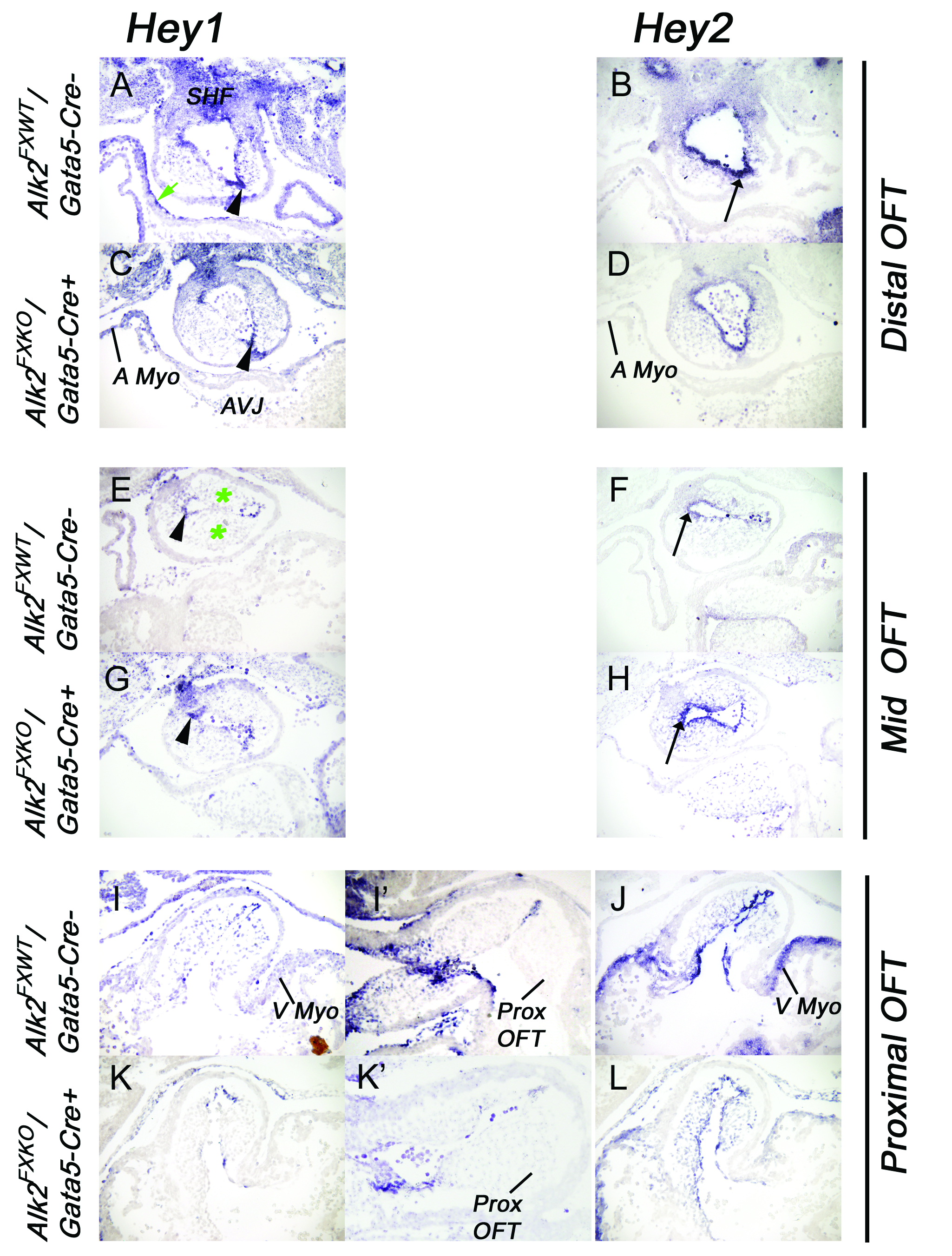

Supplement: Figure S2 — No difference in regional patterns of Notch1 target genes Hey1 and Hey2 between control and Alk2/Gata5-Cre mutant outflow tracts. ISH performed on sections transverse to distal (A–D), mid (E–H) and proximal (I–L) outflow tract of control (A, B, E, F, I, J) and mutant (C, D, G, H, K, L) embryos showing expression patterns (blue) of Hey1 (A, C, E, G, I, K) and Hey2 (ISH: B, D, F, H, J, L). Hey1 expression in OFT endocardium (arrow head) restricted to cells lateral to but not over the two main OFT cushions (parietal, septal *). ISH staining for Hey1 on sagittal sections (distal to the left) of control (I′) and mutant (K′) also not above background in mesenchymal cells of the proximal OFT cushions (Prox OFT). Hey2 detected in most OFT endocardial cells but stronger in areas lateral to the main OFT cushions (arrows). Non-coronary leaflet will develop from the intercalated cushion on the left side in these images. Hey1 also detected in atrial appendage myocardium (A Myo) and epicardium (green arrow). Hey2 also detected in ventricular wall myocardium (V Myo). (TIF) [file pone.0035539.s002.tif]

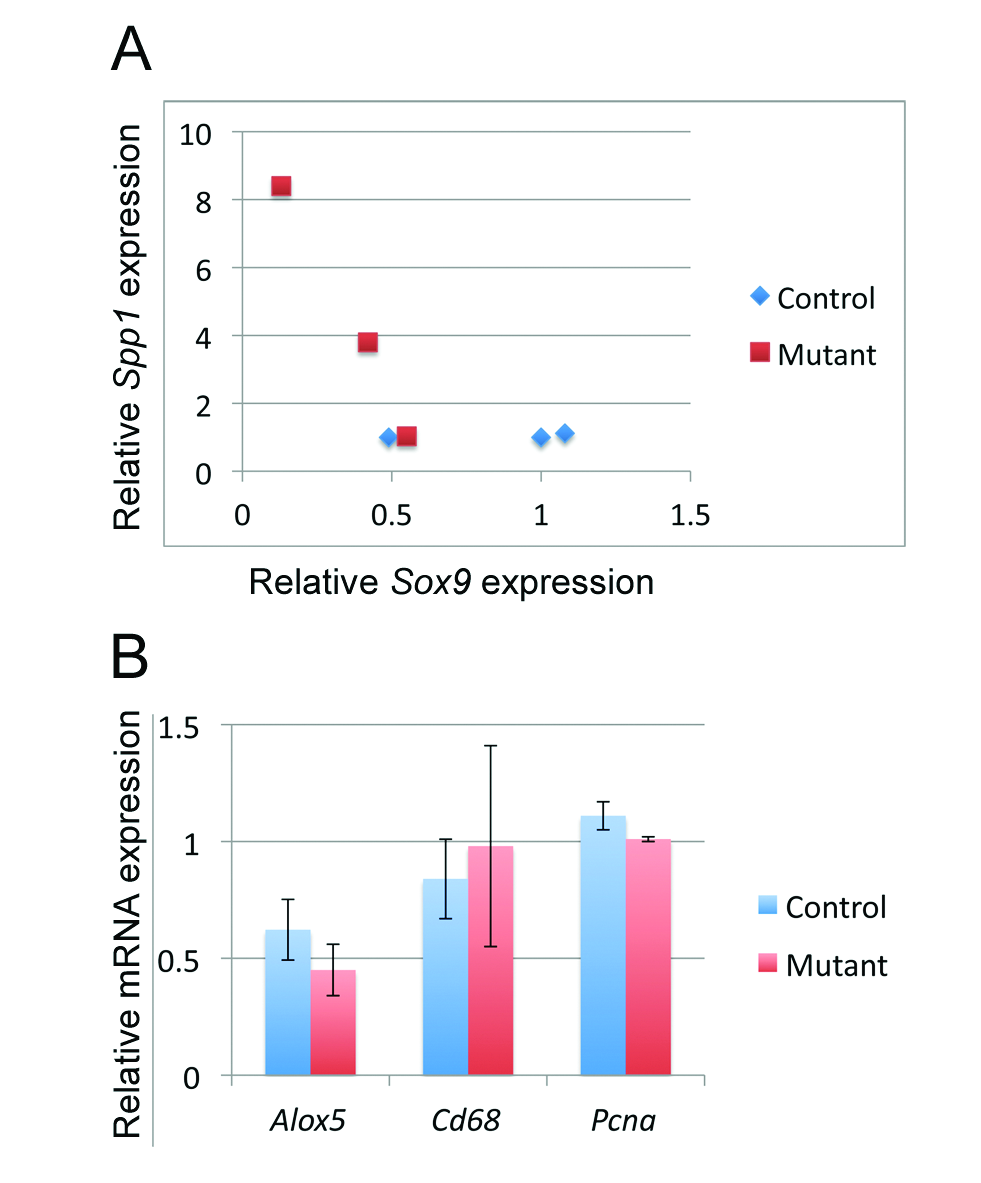

Supplement: Figure S3 — Expression findings in valve leaflets of Alk2/Gata5-Cre mutants with aortic stenosis and insufficiency. A, Scatter plot demonstrates inverse relationship between Spp1 and Sox9 expression in aortic valve leaflets of stenotic Alk2/Gata5-Cre mutants measured by real-time RT-PCR. B, Bar graph illustrates no difference in relative expression of inflammation markers Alox5 and Cd68, and of proliferation marker Pcna, in stenotic mutant (red) and normal control (blue) aortic valve leaflets measured by real-time RT-PCR quantification (n = 3). Error bars, SEM. (TIF) [file pone.0035539.s003.tif]
